# Accurate evaluation of the progress of delivery with transperineal ultrasound may improve vaginal delivery: a single-center retrospective study

**DOI:** 10.1038/s41598-023-47457-2

**Published:** 2023-11-28

**Authors:** Naosuke Enomoto, Shintaro Maki, Masafumi Nii, Mizuki Yamaguchi, Yuya Tamaishi, Sho Takakura, Shoichi Magawa, Kayo Tanaka, Hiroaki Tanaka, Eiji Kondo, Shinji Katsuragi, Tomoaki Ikeda

**Affiliations:** 1 Department of Obstetrics and Gynecology, Matsusaka Chuo General Hospital, 102 Kawaimachi, Matsusaka, Mie Japan; 2 Department of Obstetrics and Gynecology, Mie University Faculty of Medicine, Mie, Japan; 3 Department of Obstetrics and Gynecology, Faculty of Medicine, University of Miyazaki, Miyazaki, Japan

**Keywords:** Ultrasonography, Predictive markers

## Abstract

Although digital examination of the cervix is the standard method used worldwide for evaluating the progress of delivery, it is subjective. Transperineal ultrasound (TPU) is combined with digital evaluation for accurate assessment of fetal descent and rotation of the advanced part of the fetus. This retrospective study aimed to clarify the impact of introducing TPU on perinatal outcomes at Mie University Hospital. We analyzed singleton pregnant women who underwent delivery management at our hospital between April 2020 and March 2021. Perinatal outcomes were compared between patients who used TPU (TPU+ group) and those who did not (TPU− group). The angle of progression and head direction were measured. The rate of vaginal delivery was significantly increased (90.9% vs. 71.6%; P = 0.0017), and the second stage of labor was significantly prolonged in the TPU+ group (148.1 vs. 75.8 min; P < 0.0001). A significant difference was observed in termination in the latent phase between the TPU+ group [3/8 (37.5%) cases] and TPU− group [20/25 (80.0%) cases] (P = 0.036). The rate of vaginal delivery can be increased through accurate evaluation of the progress of delivery with TPU.

## Introduction

Digital examination of the cervix (DEC) is the standard method used to evaluate the progress of delivery worldwide. The advantage of DEC is that it can be performed at any time without special equipment. However, it is subjective, with a possibility of inter-examiner error. The evaluation of delivery progression plays a major role in determining the subsequent delivery method.

Transperineal ultrasound (TPU) combined with DEC is used to accurately assess fetal descent and rotation of the advanced part of the fetus. According to guidelines on labor management, TPU is not considered a standard management approach^1^. However, previous studies have reported fewer measurement errors with DEC combined with TPU than with DEC alone^2–9^, and the use of TPU has become more common in recent years. Various findings have been reported regarding the use of TPU during delivery^10–19^. Among several parameters, angle of progression (AoP) correlates best with descent of the fetal head station and low inter-examiner error^12^. For reference, AoP, considered the best indicator of head station assessment, corresponds approximately to station ± 0 at 120° of AoP, station + 2 at 140°, and station + 3 to 4 at 160°. Head direction (HD) is examined to determine the direction and extension of fetal head, whereas the midline angle confirms the position of sagittal suture and indicates the internal rotation of the head. At our hospital, we implemented TPU in labor and delivery management in April 2020. This study aimed to clarify the effects of the introduction of TPU on vaginal delivery rate.

## Materials and methods

### Study design and patients

We conducted a retrospective study to compare perinatal outcomes between patients who used TPU (TPU+ group) and those who did not (TPU− group).

### Inclusion and exclusion criteria

The inclusion criteria were as follows: (1) singleton pregnant women admitted to our delivery room for labor trials between April 2020 and March 2021, (2) term delivery, (3) primipara, and (4) availability of necessary information in medical records. The exclusion criteria were as follows: (1) age < 18 years and (2) no wish to participate in the study.

### Methods of TPU examination

TPU examinations were performed by two obstetricians (N.E. and S. Maki) in charge of delivery at our hospital. TPU was conducted immediately after each DEC by physicians. AoP and HD were measured in the TPU+ group according to established guidelines^1^. The patient assumed a semirecumbent position with legs flexed at the hips and knees at 45° and 90°, respectively, while a probe was placed between the two labia majora or more caudally, at the level of the fourchette, with an empty bladder. We used an ultrasound machine with a convex array probe (Voluson P8; General Electric, Boston, MA, USA) located in the delivery room. Patients were able to view the screen during the TPU examination. Notably, TPU examination was not performed in the TPU- group, but otherwise, the protocol for delivery management was not different between the two groups.

### Delivery management at our hospital

#### Admission to delivery

If patients experience labor onset outside the hospital, the midwife typically decides to admit these patients to the delivery room after receiving a call from them. However, in cases of emergencies, such as when patients are transported by ambulance, physicians are contacted first who tend to the patients in the emergency room. If patients’ general condition is deemed satisfactory, they are transferred to the delivery room.

#### First stage of labor

An opening of > 6 cm is considered to define the active phase of labor, and an intervention is performed if no progress in delivery is observed for 2 h in the active phase. In principle, an intrauterine manometer is inserted to measure intrauterine pressure. However, if an intrauterine manometer cannot be inserted, cardiotocography (CTG) is used to induce labor such that the number of contractions does not exceed five per 10 min. If the contractions are < 200 Montevideo units, oxytocin is used to augment labor. Additionally, the patient undergoes a cesarean section if > 200 Montevideo units are maintained for over 4 h. At our hospital, we proactively use a fetal scalp spiral electrode and insert an intrauterine manometer when CTG monitoring cannot be properly performed^20^; as a rule, the electrode is attached during the active phase^21–24^.

#### Second stage of labor

Interventions are initiated if there is no descent of the fetal head for 1 h after full dilation. When possible, an intrauterine manometer is inserted to measure the intrauterine pressure. The rotation of the fetal head is checked for abnormalities. If necessary, labor is augmented when contractions are weak; however, mechanical delivery is performed when the requirement for mechanical delivery is met. If the indications for instrumental delivery have not been met after 3 h have elapsed, and weak pain has been overcome, a cesarean section is performed. Regarding delivery effort, the “delayed push” method is typically used^25^.

#### Induction of labor

For patients with Bishop scores of < 4 points without premature rupture of membranes (PROM), we suggested mechanical or pharmacological cervical ripening methods for labor induction, and patients selected their preferred method. In cases of PROM without labor onset, procedures for cervical ripening are avoided due to the risk of infection. Instead, oxytocin induction is performed, regardless of the Bishop score.

#### Epidural anesthesia

The obstetricians in charge of delivery also perform epidural anesthesia. Analgesics are administered intermittently every hour, typically using lumbar (L) 2/3 or L3/4 as the puncture point. The anesthesia consists of 6–10 mL of the drug mixture, which includes 7 mL of 0.1% ropivacaine (7.5 mg/mL) + 2 mL of fentanyl (0.1 mg/1A) + 41 mL of saline solution, is administered as a rule. The aim is ''walking anesthesia,'' while slightly preserving the motor nerves. Augmentation with oxytocin is always considered along with starting anesthesia.

#### Vacuum delivery

Vacuum delivery is restricted to no more than five times and a maximum duration of 20 min from the initial vacuum application^23,24,26^.

#### Cesarean section

The decision for a cesarean section was based on the response after an active phase, as described in the *first and second stages of labor* sections above. In cases of an abnormal latent phase of labor, our goal was delivery within 3 days after termination was decided. For patients with Bishop scores of < 4 points, we performed mechanical or pharmacological cervical ripening methods for labor induction. We also considered using oxytocin for patients with Bishop scores of > 4 points. Amniotomies were performed occasionally. If the latent phase showed no progression, we performed a cesarean section. Cases other than those of labor arrests, such as non-reassuring fetal status (NRFS) and hypertensive emergencies, were managed on a case-by-case basis.

### Statistical methods

Statistical analysis was conducted using Fisher's exact, Mann–Whitney U, and the χ-square tests, with P-values used for comparing maternal characteristics. Statistical significance was considered at P < 0.05. All statistical analyses were performed using JMP PRO 14 software (SAS Institute Inc, Cary, North Carolina).

### Ethics approval

This study was conducted in accordance with the principles of the Declaration of Helsinki and approved by the Institutional Review Board of Mie University Hospital (approval number: H2023-012, January 23, 2023). The need to obtain informed consent from the study participants was waived by the Institutional Review Board of Mie University Hospital since this is a retrospective analysis. Patients who were eligible for this study had the opportunity to refuse to participate in the study by opting out.

## Results

Table 1 shows the characteristics of patients in the TPU+ and TPU− groups. No differences were found in the rates of pre-pregnancy complications, including cardiac, liver, renal/urinary tract, endocrine, psychiatric, and gynecologic issues; thrombosis/embolism; inflammatory bowel disease; severe obesity (BMI > 35 kg/m^2^); and orthopedic disease. Significant differences were observed in obstetric complication rates (P < 0.0001). Obstetric complications included 12 cases of gestational diabetes mellitus (GDM), four hypertensive disorder in pregnancy (HDP), two fetal growth restriction (FGR), one abruption of the placenta, and one polyhydramnios, respectively, in the TPU+ group (duplicates included). In contrast, nine cases of HDP, eight FGR, seven GDM, five PROM, three oligohydramnios, two abruption of the placenta, two chorioamnionitis, one polyhydramnios, and two others were observed in the TPU− group (duplicates included). A significant difference was found in the rates of malrotation (P = 0.028) and epidural anesthesia (P = 0.031).

**Table 1:** Characteristics of patients in both groups.

	TPU+	TPU−	P-value
Case	88	88	–
Age^a^	33.8 ± 5.4	31.2 ± 5.9	NS
Pre-pregnancy BMI > 25 kg/m^2^	47 (53.4%)	24 (27.3%)	0.0017
Pre-pregnancy complication	49 (55.7%)	40 (45.5%)	NS
Obstetric complication	17 (19.3%)	50 (56.8%)	< 0.0001
Malrotation^b^	12 (13.6%)	3 (3.4%)	0.028
Transported by ambulance^c^	5 (5.7%)	4 (4.5%)	NS
Admission days^a^	263.9 ± 46.1	268.8 ± 21.3	NS
Bishop score at admission^a^	2.7 ± 2.7	2.7 ± 3.1	NS
Oxytocin for induction of labor^d^	8 (9.1%)	11 (12.5%)	NS
Metreurynter for induction of labor^d^	5 (5.7%)	10 (11.4%)	NS
Controlled-release dinoprostone vaginal delivery system for induction of labor^d^	25 (28.4%)	30 (34.1%)	NS
Oxytocin for augmentation	60 (68.2%)	50 (56.8%)	NS
Epidural anesthesia^e^	15 (17.0%)	5 (5.7%)	0.031

*TPU* transperineal ultrasound, *BMI* body mass index, *min* minutes, *PROM* premature rupture of membrane, *NS* not significant.

^a^Mean ± standard deviation.

^b^One case of TPU+ delivered in face presentation and the rest delivered in occiput posterior position.

^c^Patients were transported by ambulance from another clinic after labor onset or PROM.

^d^We selected oxytocin for cases of PROM or those with Bishop score > 4 points. For cases with Bishop score < 4 points, we presented the metreurynter or controlled-release dinoprostone vaginal delivery systems, and patients chose which to use. Spontaneous labor onset occurred in 50 patients in the TPU+ group and 37 in the TPU− group (p = NS).

^e^Anesthesia was performed at the patient's request in all cases of this study.

Table 2 shows the outcomes in both groups. The rate of vaginal delivery was significantly increased, and the second stage of labor was significantly prolonged in the TPU+ group.

**Table 2:** Outcomes in both groups.

	TPU+ (n = 88)	TPU− (n = 88)	P-value
Delivery days (days)^a^	278.2 ± 7.9	276.8 ± 8.6	NS
Birth weight (g)^a^	3090.3 ± 437.9	2958.9 ± 444.4	NS
Blood loss > 1000 g	28 (31.8%)	20 (22.7%)	NS
Apgar score 1 min^a^	7.9 ± 0.9	7.9 ± 1.0	NS
Apgar score 5 min^a^	8.9 ± 0.6	8.8 ± 0.7	NS
Umbilical artery pH^a^	7.27 ± 0.07	7.27 ± 0.08	NS
Vaginal delivery rate	80 (90.9%)	63 (71.6%)	0.0017
The second stage of delivery (min)^a^	148.1 ± 107.7	75.8 ± 88.8	< 0.0001

*min* minutes, *NS* not significant, *TPU* transperineal ultrasound.

^a^Mean ± standard deviation.

The reasons for emergent cesarean section in both groups are shown in Table 3. No significant differences were found in each category of indication. Termination in the latent phase occurred in 3/8 (37.5%) and 20/25 (80.0%) cases in the TPU+ and TPU− groups, respectively, with significant differences (P = 0.036).

**Table 3:** Reasons for emergency cesarean section and timings in both groups.

	TPU+ (n = 8/88)	TPU− (n = 25/88)	P-value
Failure of vacuum delivery	1/8 (12.5%)	0/25 (0%)	NA
Arrest of labor	5/8 (62.5%)	10/25 (40.0%)	NS
NRFS	1/8 (12.5%)	10/25 (40.0%)	NS
Others^a^	1/8 (12.5%)	5/25 (20.0%)	NS
**Termination at the latent phase**	3/8 (37.5%)	20/25 (80.0%)	0.036

*NRFS* non-reassuring fetal status, *HDP* hypertensive disorder in pregnancy, *NS* not significant, *NA* not applicable, *TPU* transperineal ultrasound.

^a^One case of HDP in the TPU+ group and one of HDP, one of positional abnormalities during ongoing labor, one of advanced umbilical cord, one of intrauterine infection, and one of abruption in the TPU− group.

**Figure 1:**
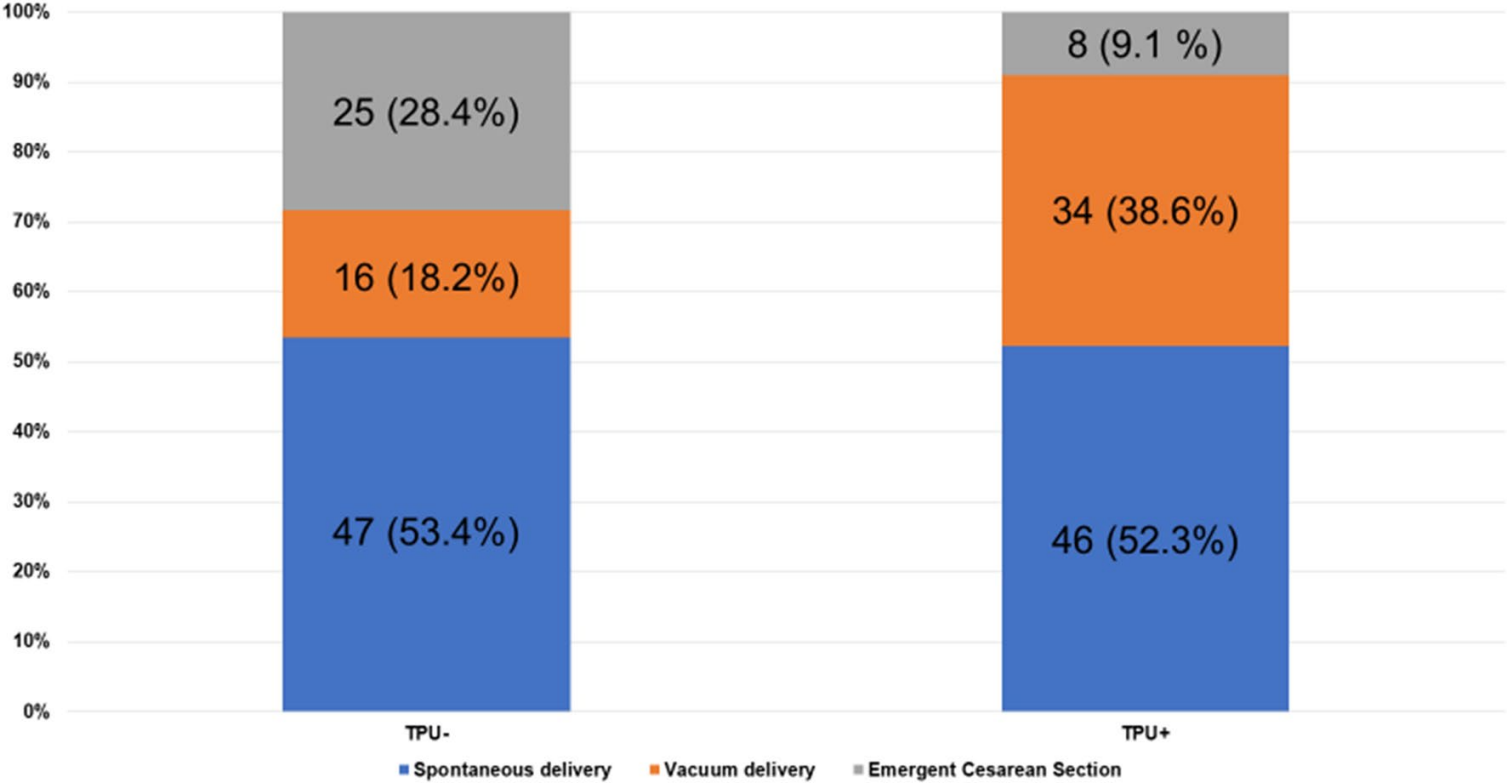
The number of cases (percentage) of the mode of delivery.

As shown in Fig. 1, vacuum deliveries accounted for 38.6% and 18.2%, spontaneous deliveries for 52.3% and 53.4%, and emergent cesarean sections for 9.1% and 28.4% of TPU+ and TPU− deliveries, respectively. An increase in vacuum deliveries and a decrease in emergent cesarean sections were also observed.

**Figure 2:**
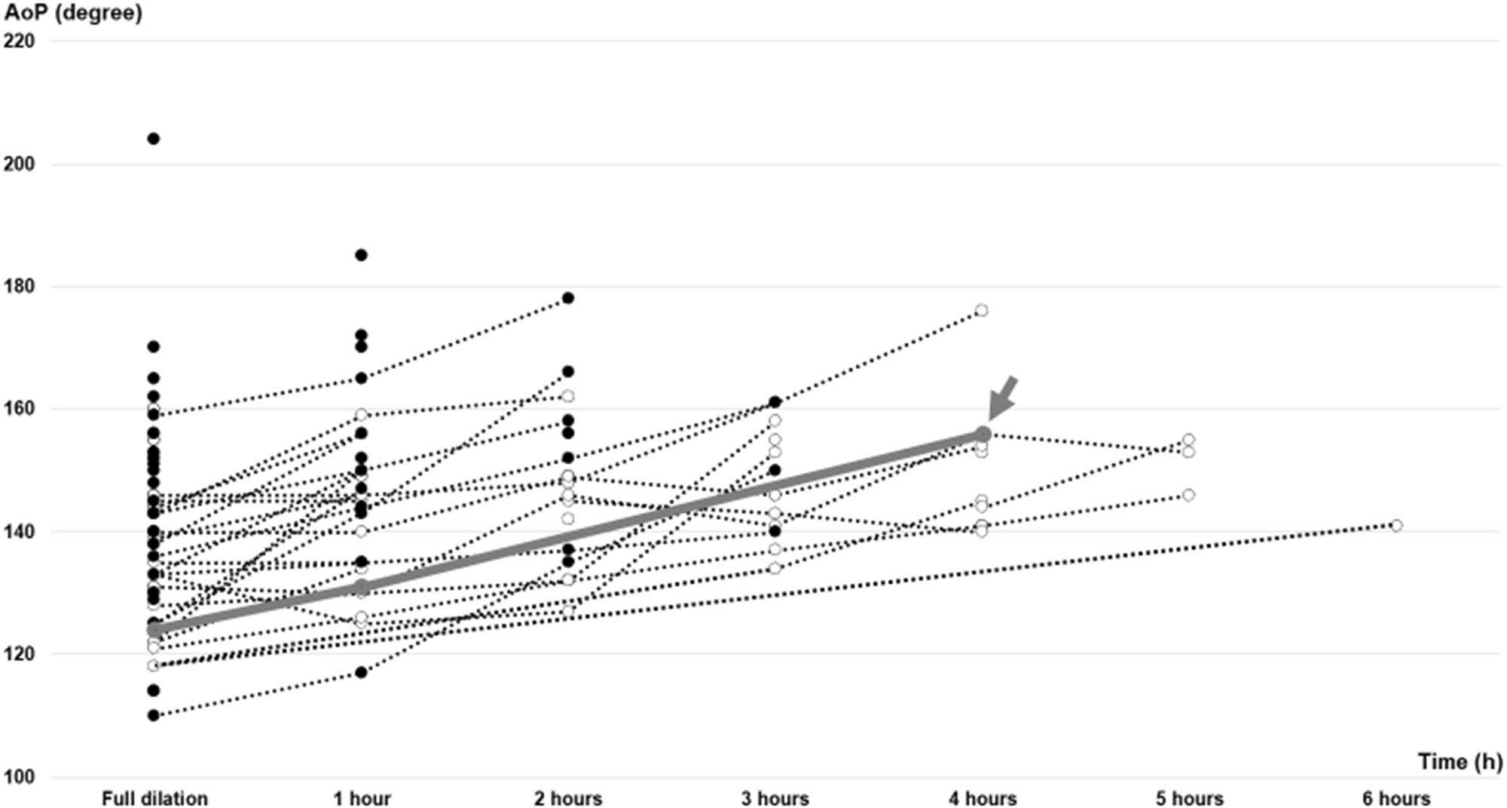
The trends of change in AoP in the TPU+ group. This graph depicts the plots of the measurement of the TPU+ group. Black dots represent vaginal delivery cases, and white dots represent vacuum cases. One patient, illustrated by the thick solid line, underwent an emergent cesarean section after a failed vacuum delivery at the arrowed point.

Figure 2 shows the trends of change in AoP in the TPU+ group. The average AoP among the vacuum cases was 151°. A patient who underwent an emergent cesarean section after a failed vacuum delivery had no rotation abnormalities, and the HD was upward. This case was vacuumed at 156°. Furthermore, the remaining seven patients in whom cesarean sections were performed underwent the procedure before full dilation and are not plotted in this graph.

**Figure 3:**
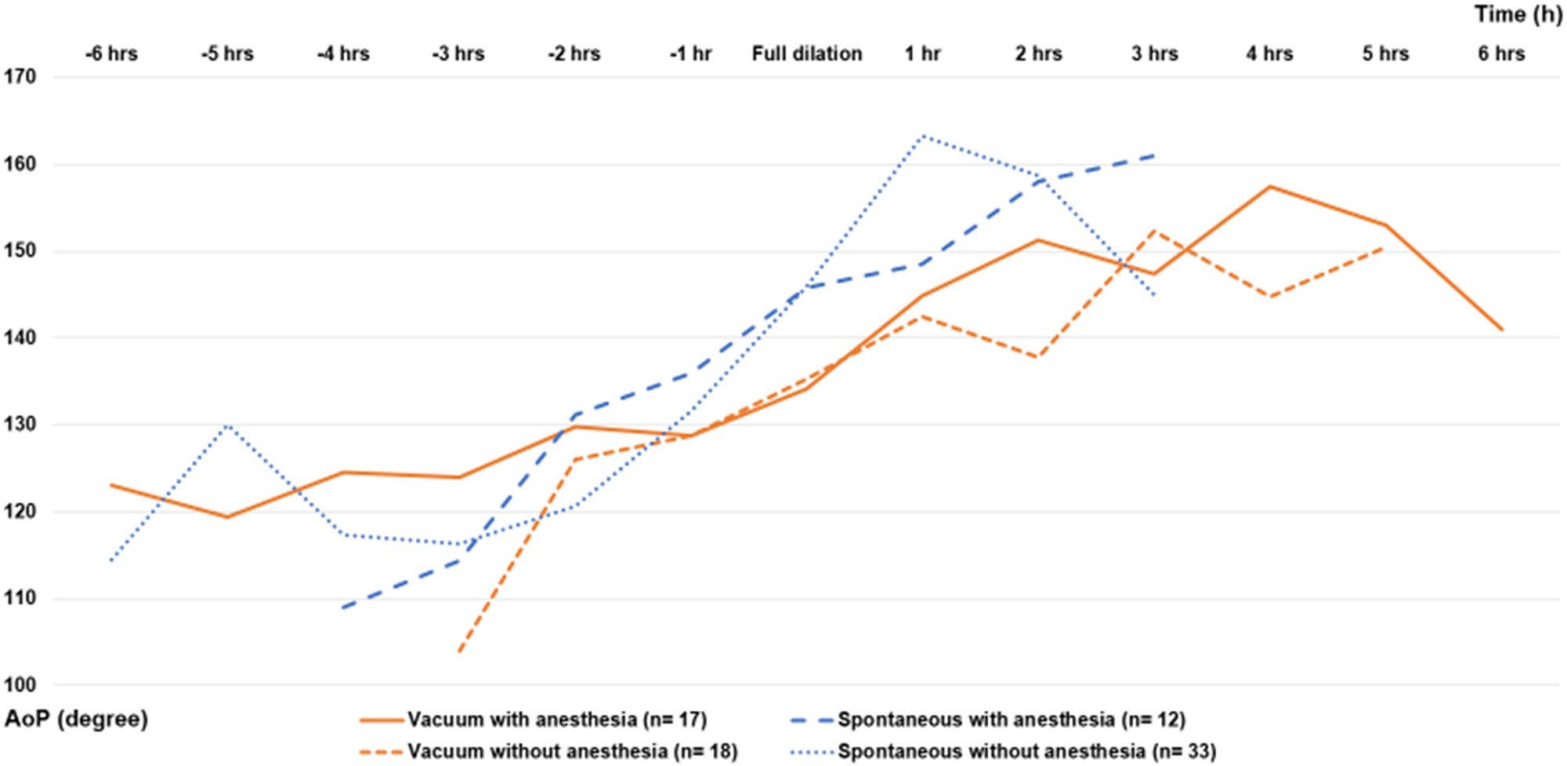
The trends of change in AoP of spontaneous or vacuum delivery with/without anesthesia. This is a graph of hourly changes measured with the center of the horizontal axis at the point of full dilation. The vertical axis represents the angle of AoP.

As shown in Fig. 3, the AoP trend was slower in patients who underwent vacuum delivery than in those who underwent vaginal delivery.

## Discussion

This study examined the impact of introducing TPU on perinatal outcomes, and the results suggest that TPU, which is an accurate and objective method, may have improved the assessment of the progress of delivery and increased the rate of vaginal delivery. We believe this is due to decreased underestimation of the delivery process. This improvement is particularly noteworthy in a high-risk hospital setting, such as our hospital. Among the 176 participants enrolled in this study, 89 (50.5%) and 67 (38.1%) had pre-pregnancy and obstetric (including overlap) complications, respectively. This indicates the significance of the impact of the study's results, which is the increased rate of vaginal delivery at a high-risk center.

In Japan, mechanical cervical ripening methods, including a metreurynter, are mainly used for cervical ripening. However, the approval of the controlled-release dinoprostone vaginal delivery system (PROPESS) by the Ministry of Health, Labor, and Welfare in January 2020 provided an alternative with a similar success rate for labor induction. Previous studies have reported that the success rate of labor induction of PROPESS is similar to that of mechanical cervical ripening methods.^27,28^ For PROM cases, we used oxytocin for induction. In this study, no significant differences were found in the labor induction methods.

In this study, patients were assigned to the TPU+ or TPU− group when they entered the delivery room. Therefore, we considered that the increased rate of vaginal deliveries extended beyond cases involving labor arrest. As shown in Table 2, the observed prolongation of the second stage of labor implies that physicians occasionally underestimated the progress of labor, which led them to diagnose labor arrest without TPU. This underestimation was attributed to the challenges in diagnosing the head station during DEC, particularly due to the appearance of a caput succedaneum rather than cervical dilation. Therefore, the condition in some cases may have been diagnosed as labor arrest when no change was observed in cervical dilation, particularly after full dilation. According to the Recommendations for the Safe Prevention of the Primary Cesarean Delivery^24^, before diagnosing labor arrest in the second stage, at least 3 h of pushing in nulliparous women should be allowed if the maternal and fetal conditions permit. In addition, longer durations may be appropriate on an individualized basis as long as progress is documented. This study emphasized the importance of using TPU during the second stage of labor for accurate assessment of the progress of delivery. Emergent cesarean sections after full dilation decreased from 9/189 cases in 2019 to 2/192 cases in 2020, although without significant difference (Supplementary Fig. 1A, B). Avoiding emergent cesarean section after full dilation can potentially result in better perinatal outcomes and reduced negative impacts on subsequent pregnancies^27–30^. However, since no association was found between TPU and cesarean indications, further studies are needed to investigate the underestimation of labor, particularly by focusing on cases of labor arrest.

For the active phase, we managed to measure intrauterine pressure in 37 cases (Supplementary Fig. 2) but could not achieve measurements exceeding > 200 Montevideo units in other cases. Subsequent studies should aim to measure intrauterine pressure in all cases to gain a comprehensive understanding. A significant difference was found between the TPU+ and TPU− groups in judging during the latent phase, indicating the usefulness of accurate assessment of the descent of the fetal head.

Furthermore, the duration of the second stage of labor may be affected by the increasing use of epidural anesthesia^30^. Prior research has shown that the duration of the second stage of labor^31,32^ tends to increase with epidural anesthesia administration, leading to higher rates of instrumental delivery^33,34^ and weak contractions^35,36^. Some reports indicate that rate of cesarean section remains the same^31,37^, whereas others suggest that the rate increases^38,39^. We think the increased rate of malrotation in our study may be due to anesthesia^40–42^.

At our hospital, we routinely consider augmentation with oxytocin along with initiation of anesthesia. In this study, oxytocin was combined with initiation of epidural anesthesia in most cases. Therefore, we created Fig. 3 to examine the effect of anesthesia on the second stage of labor in this study. The χ-square test was performed on the slope of the AoP from initiation of full dilation to 2 h afterward, and no significant difference was found regarding the effect of anesthesia.

For patients who underwent spontaneous vaginal delivery, the χ-square test showed a difference in the AoP slope in the second stage of labor (P = 0.0004) compared with that noted in patients who underwent vacuum delivery. These results suggest that patients requiring vacuum delivery had slower progress of delivery, regardless of the use of anesthesia.

As shown in Supplementary Fig. 2, patients in whom intrauterine pressure was measured consistently showed higher intrauterine pressure in patients who received anesthesia, leading to spontaneous labor. While a decrease in uterine contractility has been shown in previous reports^35,36^, the present study shows that appropriate use of uterine contractions may prevent weak contractions.

Regarding the head station in the patients who underwent vacuum delivery, as shown in Fig. 3, the average AoP during the full dilation was 135°, whereas that at 1, 2, 3, 4, 5, and 6 h after full dilation was 143°, 143°, 149°, 151°, 151°, and 141°, respectively. These mean changes represent gradual progression in AoP over time (several degrees per hour), which could be challenging to diagnose accurately using digital evaluation alone. However, small changes in AoP measurement could be considered within the error range. Therefore, combining multiple ultrasound findings, such as a change in AoP at a push^29^, HD, and midline angle^10–19^, would support this concern. Moreover, detection of subtle changes in AoP that might go unnoticed by manual examination could prevent unnecessary cesarean sections. In the patients who delivered spontaneously, the average AoP at full dilation was 145°, and that at 1, 2, and 3 h after full dilation was 160°, 159°, and 150°, respectively. The declining angles are likely due to the increasing number of cases of delivery. Although vacuum deliveries were more frequent in the TPU+ group, no subgaleal hematoma or cerebral hemorrhage was observed in any vacuum deliveries performed in this study. Instead, mild cephalhematoma occurred in four cases in the TPU+ group and two cases in the TPU− group, while scalp epidermolysis was observed in one case in both the groups.

However, this study did not determine the prevention of delivery overestimation, that is, increased cesarean section due to failed instrumental delivery. As shown in Table 3, only one TPU+ case of failed vacuum delivery was present. While several reports exist on mechanical deliveries^43–47^, further accumulation of cases is warranted to determine the possibility of reducing the overestimation of deliveries.

Regarding clinical practices at our hospital, we hold a conference every morning to review the CTGs of deliveries as part of the education of the residents; however, we could not review DEC findings. The AoP values allow us to assess the degree of head station retrospectively and provide more practical feedback. The residents are trained to evaluate the degree of the head station through digital evaluation with senior doctors. With the use of ultrasound, resident physicians are making faster progress at present than in previous years because they provide objective numerical results. Additionally, ultrasound images can serve as useful communication tools for decision-making in the delivery room for midwives, nurses, and patients.

This study has some limitations. First, this was a retrospective study, and no significant difference was found in the percentage of labor arrest, probably due to the sample size. Second, a significant difference was noted in the rate of anesthesia use; therefore, the impact of this difference cannot be ignored. Third, as shown in Supplementary Fig. 1A, B, the rate of vaginal deliveries increased significantly from 2019, when no TPU was performed, to 2020, when this study commenced. This suggests that the introduction of TPU may have contributed to overall improvements in digital evaluation techniques at our hospital. If so, the effect of improving DEC techniques throughout the facility on the TPU- group cannot be ruled out. Discrepancies in the measurement intervals were another limitation. In this study, we commenced AoP measurement when the patient entered the delivery room and measured it at each DEC, indicating the need to standardize the starting point of AoP measurement. Finally, there might be a bias in case selection, given that more cases with obstetric complications were found in the TPU− group, which may have been affected by the increased number of cesarean sections. Considering these factors, a prospective study should be conducted in the future to further investigate these findings.

## Conclusion

This study demonstrates that TPU may increase the rate of vaginal delivery. Accurate evaluation of the descent of the fetal head station may prevent underestimation of the progress of delivery and decrease the frequency of emergency cesarean sections. Moreover, TPU evaluation can be used as a basis for retrospective reviews, including CTGs, and offer valuable feedback as feedback for educational purposes in clinical settings.

### Supplementary Information

Supplementary Figure 1.

Supplementary Figure 2.

Supplementary Figure 3.

Supplementary Legends.

## Data Availability

The data that support the findings of this study are available on request because the data contain potentially identifying or sensitive patient information. This restriction is imposed by the Institutional Review Board (Contact; Data Manager: imaiya@med.mie-u.ac.jp).
