# Supplementary figures and images for: Accurate evaluation of the progress of delivery with transperineal ultrasound may improve vaginal delivery: a single-center retrospective study
Source: Sci Rep. 2023 Nov 28;13:20945. doi: 10.1038/s41598-023-47457-2 (PMC10684555; doi:10.1038/s41598-023-47457-2)

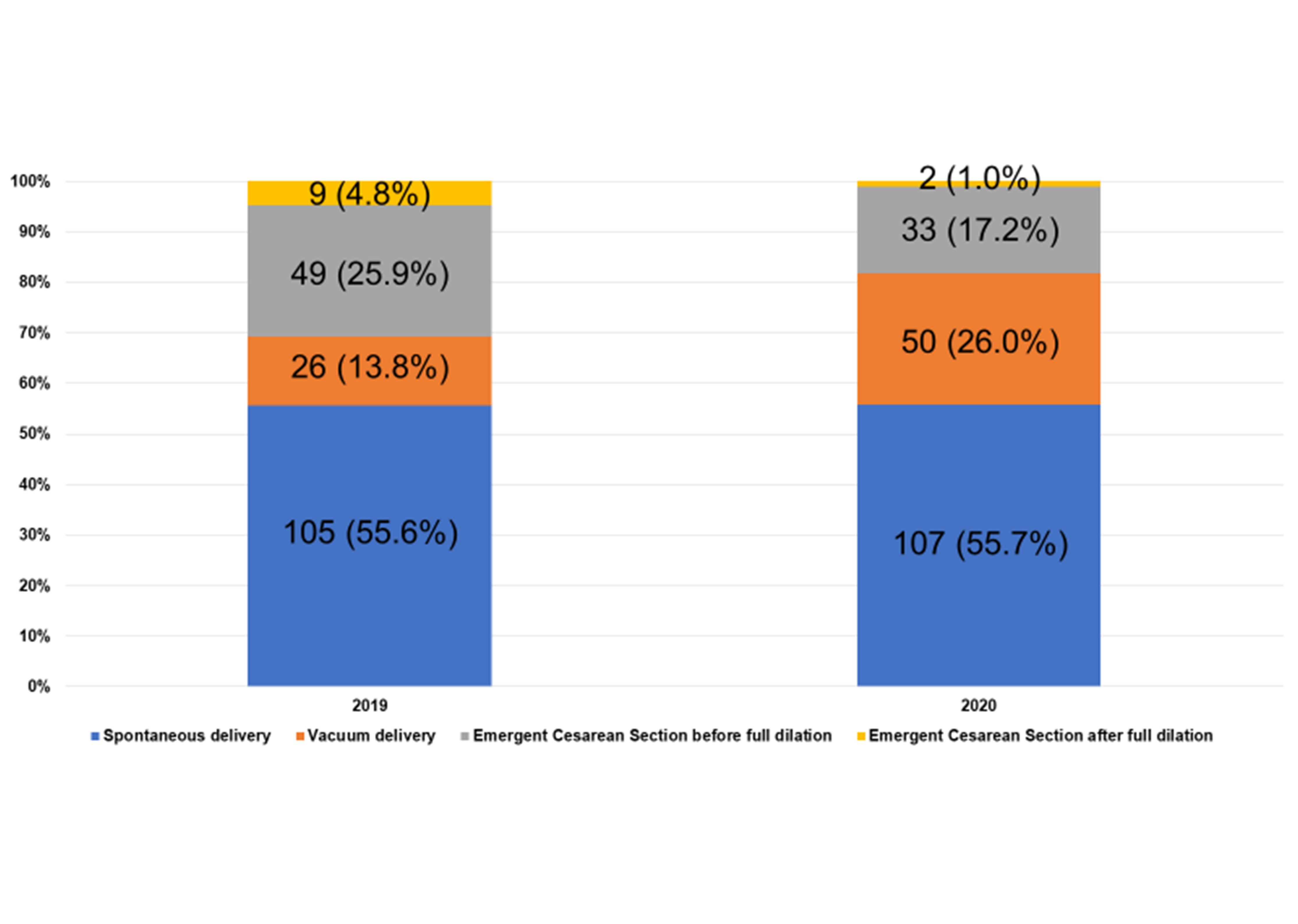

Supplement: Supplementary file 1 — Supplementary Figure 1. [file 41598_2023_47457_MOESM1_ESM.jpg]

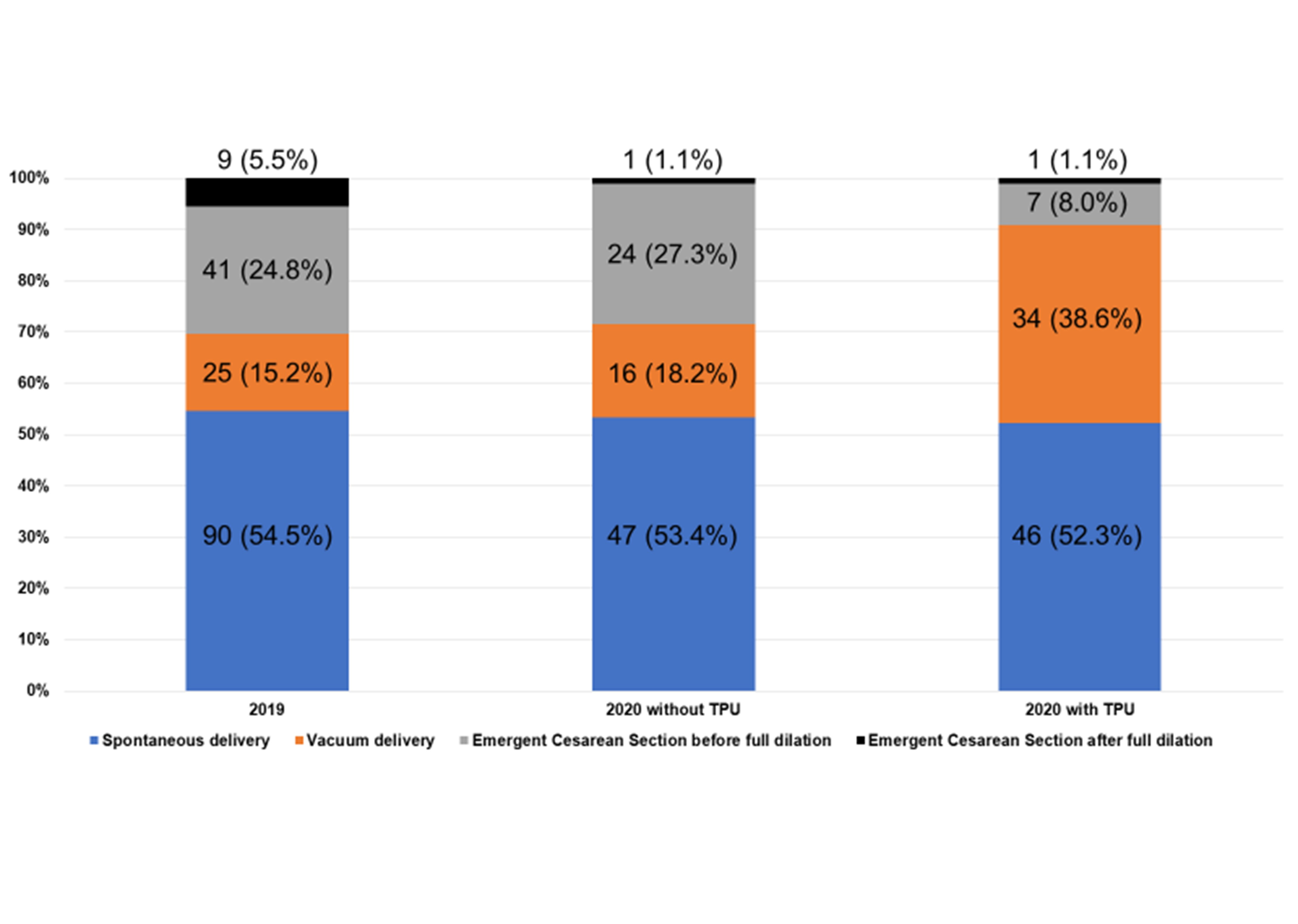

Supplement: Supplementary file 2 — Supplementary Figure 2. [file 41598_2023_47457_MOESM2_ESM.jpg]

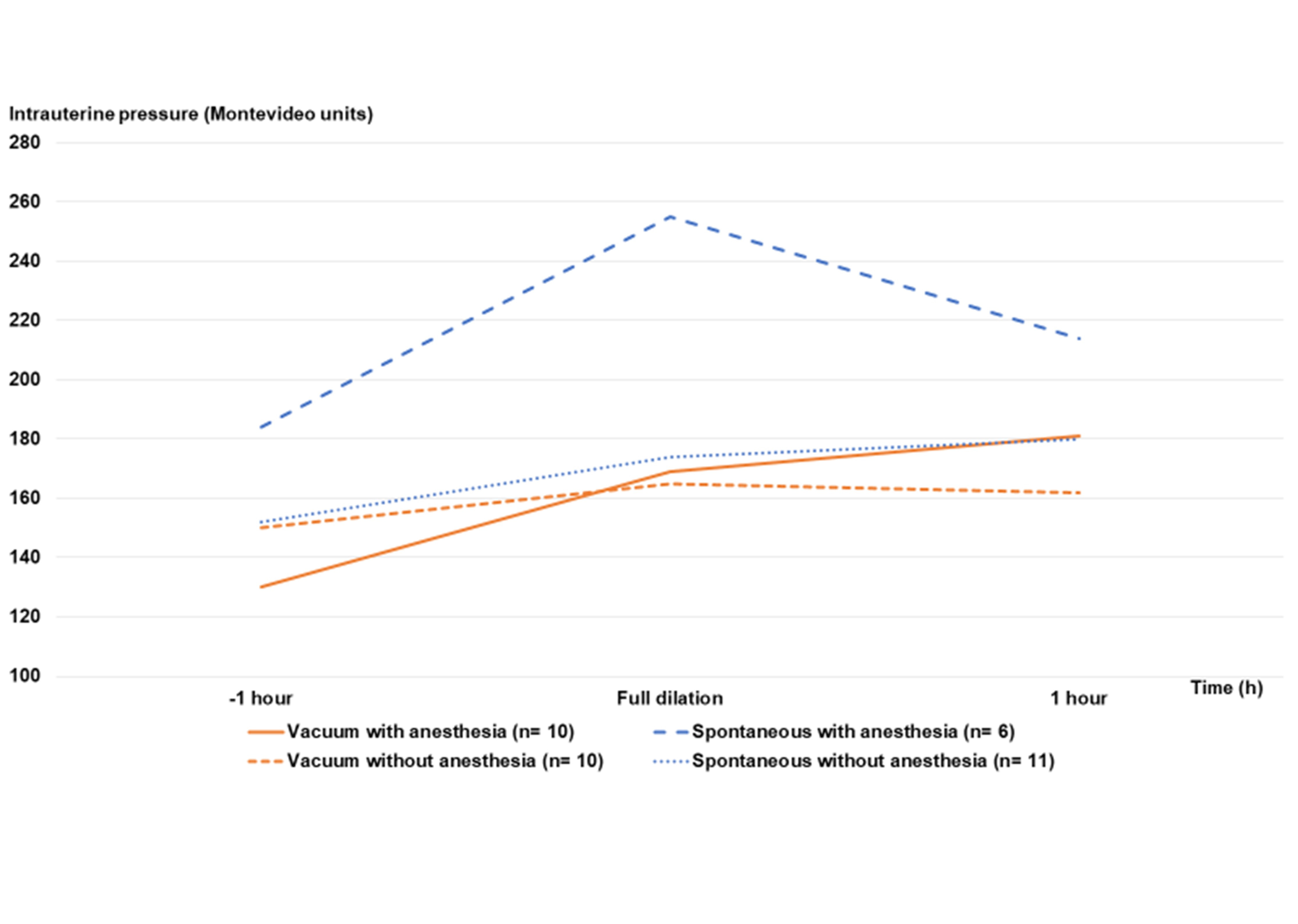

Supplement: Supplementary file 3 — Supplementary Figure 3. [file 41598_2023_47457_MOESM3_ESM.jpg]
