# Supplementary material for: Accurate evaluation of the progress of delivery with transperineal ultrasound may improve vaginal delivery: a single-center retrospective study
Source: Sci Rep. 2023 Nov 28;13:20945. doi: 10.1038/s41598-023-47457-2 (PMC10684555; doi:10.1038/s41598-023-47457-2)
Supplement: Supplementary file 4 — Supplementary Legends. [file 41598_2023_47457_MOESM4_ESM.docx]

**Supplementary Material**

**Supplementary Figure 1A.** This graph shows that the rate of vaginal deliveries increased from 2019 to 2020. The graphs show the mode of delivery in 2019 and 2020 including preterm birth in primiparous women. Transvaginal and vacuum deliveries increased.

**Supplementary Figure 1B.** This graph shows the mode of delivery in 2019, 2020 without TPU, and 2020 with TPU. When separated with or without TPU, the graph compositions of 2020 without TPU and 2019 were similar.

**Supplementary Figure 2.** This graph shows the average intrauterine pressure. The horizontal axis represents time, and the vertical axis represents the Montevideo units. The group that had epidural anesthesia and delivered spontaneously had higher intrauterine pressure than the other groups.
